# Measuring (Mis)trust in the age of COVID-19: viewpoints of vaccine clinical trial participation among individuals living with sickle cell disease

**DOI:** 10.1186/s12889-025-22731-2

**Published:** 2025-04-28

**Authors:** Khadijah Abdallah, Kiana Amini, Hasmin C. Ramirez, Madison Keller, Diba Seddighi, Marilyn S. Baffoe-Bonnie, Shameka Thomas, Vence L. Bonham, Ashley J. Buscetta

**Affiliations:** 1 Social and Behavioral Branch, National Human Genome Research Institute, National Institutes of Health, Bethesda, MD US; 2 Rollins School of Public Health, Emory University, Atlanta, GA US; 3 School of Medicine, Louisiana State University, New Orleans, LA US; 4 Department of Bioethics, National Institutes of Health, Bethesda, MD US; 5 Department of Sociology, Rutgers University, New Brunswick, NJ US; 6 Center for Bioethics, The Ohio State University College of Medicine, Columbus, OH US

**Keywords:** COVID-19, Institutional medical mistrust, Chronic diseases, Sickle cell disease, Vaccines, Clinical trials

## Abstract

**Background:**

COVID-19 vaccine efficacy was determined by the participation of individuals from diverse backgrounds in clinical trials. While these trials recruited participants with chronic conditions, little is known about how sentiments of mistrust affected the views of vaccine trial participation. The aim of this study is to examine the relationship between self-reported institutional medical mistrust and views on the importance and utility of COVID-19 vaccine research participation among adults living with sickle cell disease (SCD) in the United States.

**Methods:**

This cross-sectional study is part of a larger longitudinal study aimed at understanding the lived experiences of individuals living with SCD in the United States during the COVID-19 pandemic. Data from the first wave of the longitudinal study, collected June– December 2020, were used for the analyses (*n* = 185). Two single-item variables ascertaining the utility of participation in clinical trial research for COVID-19 vaccines were examined. Institutional medical mistrust was measured utilizing a modified medical mistrust index. Multivariable binary logistic regression models were utilized, adjusting for applicable sociodemographic, behavioral, and clinical variables.

**Results:**

A majority of study participants agreed with statements regarding beliefs about the importance of participating in vaccine research (71.4%) and the utility of vaccine research for family and self (60.0%). Findings indicated that having any level of worry of COVID-19 infection was significantly associated with greater agreement with the importance of participating in COVID-19 vaccine research (OR = 3.41, 95% CI 1.346–8.641, *p* = 0.01) and higher agreement with the utility of vaccine research for themselves and their families (OR = 3.54, 95% CI: 1.332–9.403, *p* = 0.01), after adjusting for covariates. Agreement with the utility of vaccine research participation was also found to be associated with higher SCD severity (OR = 1.26, 95% CI: 1.043–1.537, *p* = 0.02). In contrast, higher medical mistrust was inversely associated with agreement of this statement (OR = 0.44, 95% CI: 0.222–0.89, *p* = 0.02).

**Conclusions:**

Our findings reveal that for individuals living with sickle cell disease, the worry of infection and the severity of their individual disease were more important in shaping views towards vaccine research participation than medical mistrust.

## Background

The COVID-19 pandemic in the United States required a response which was led by government and healthcare sectors. The rapid spread of the disease was paralleled by the rapid development of vaccines and therapeutics. Initially, limited supply, availability, and access to vaccinations were driving forces of vaccine uptake. As supply eventually reached demand, it became more apparent that some of the United States population was hesitant and unwilling to accept the vaccine [1–4] and the introduction of new boosters continues to be a factor contributing to inconsistent vaccine uptake. As of October 2023, 230 million people (69.5% of the total U.S. population) completed their primary vaccination series, while only 56 million (17% of the total U.S. population) received a bivalent booster dose [5].

Moving vaccines from bench to arm was accomplished through rigorous research and willing clinical trial vaccine research participants. Many companies, governments, and academic research groups initiated and continue to conduct vaccine trials in response to the dangerous spread of novel variant strains of SARS-CoV-2 [6, 7]. Of importance has been successfully recruiting participants who are representative of diverse clinical populations, including individuals who are (a) high-risk for infection (e.g., frontline workers, essential workers), (b) high-risk for severe complication or death due to COVID-19 (e.g., due to age and/or co-morbidities), and (c) representative of racial, ethnic, and ancestral diversity [8, 9].

Medical mistrust due to sociohistorical factors has been a long-term barrier to trial participation and vaccine uptake [10]. Mistrust has been defined as the “general sense of unease or concern that a provider or [organization] may not act in a person’s best interest” [11]. The prevailing sense of mistrust relating to the COVID-19 pandemic has had a notable impact on the U.S. public, including affecting the behaviors of communities of color [12–14]. This mistrust throughout communities of color has been attributed to existing inequities that have been magnified by the COVID-19 pandemic and a greater loss of confidence in institutions [15–17].

Despite the growing body of literature examining the national population’s mistrust of biomedical research and institutions during the COVID-19 pandemic, there are limited empirical studies that examine how the context of living with a chronic illness may impact medical mistrust of health care-related institutions [18–21]. We aim to fill this gap by examining the relationship between institutional mistrust and views on vaccine research participation in a group of adults living with sickle cell disease (SCD).

Sickle cell disease is a group of rare inherited red blood cell disorders that can affect nearly every organ system in the body and may result in a lower life expectancy [22]. The health complications associated with SCD put individuals living with this disease at higher risk of severe complications and death due to COVID-19 [23]. In the United States, SCD predominantly affects individuals of African descent [22] and given the population’s well-documented experiences of racialized discrimination, SCD is a strong case to explore the question of how mistrust of biomedical research and/or government institutions shape views towards COVID-19 vaccine research participation [24, 25]. Furthermore, the health care views, decisions, and experiences of individuals living with SCD are often understood and reflective of the historical mistrust of biomedical research and government institutions that exists within Black and African American communities at large [26]. However, there are few studies that pointedly examine mistrust within the context of living with SCD [27, 28] and few on how mistrust affects clinical research participation in this population [26, 29, 30].

The aim of this study is to examine the relationship between self-reported institutional medical mistrust and views on the importance and utility of COVID-19 vaccine research participation among adults living with sickle cell disease (SCD) in the United States. We hypothesize that medical mistrust will be significantly associated with negative views towards COVID-19 vaccine research participation among adults with SCD.

## Methods

### Study procedure and recruitment

This is a cross-sectional study that is part of a larger longitudinal study aimed at understanding the lived experiences of individuals living with SCD in the United States during the COVID-19 pandemic. Participants were recruited through social networks, snowball sampling, and previous enrollment in an ongoing SCD study called the INSIGHTS Study (NCT02156102, approved 16/06/2014). Participants were first surveyed in June 2020, with four waves of data collection completed; the final survey was administered April 2022. Inclusion criteria for the study were: (1) adults aged 18 or older, (2) diagnosis of SCD and knowledge of type of SCD, and (3) currently residing in the United States. The web-based survey was administered using a Qualtrics platform, through which each participant was provided a unique URL survey link. Data collected for the first wave of the study were utilized for this cross-sectional analysis. Data collection occurred between June 2020 and December 2020 when the initial COVID-19 vaccines were still being tested in clinical trials and considered for emergency-use authorization by the FDA. A total of 185 eligible individuals were included in this study. Study approval was obtained through the National Institutes of Health (NIH) Institutional Review Board for protocol number 20HGN125 (NCT04417673, approved 02/06/2020).

### Outcomes

Two single-item variables with the aim of gathering perspectives regarding participation in clinical trial research for COVID-19 vaccines were used as our outcome variables. Using a five-point Likert-scale ranging from ‘strongly disagree’ to ‘strongly agree’, participants were asked to report their level of agreement with the following two items: (1) “It is important for people to take part in COVID-19 vaccine research” and (2) “Participation in COVID-19 vaccine research can help my family and me”. Both items were adapted from the measure on Perceptions of Participation in Clinical Research [31] and were modified for COVID-19. For the purposes of this study, responses were classified as either agreeing (agree or strongly agree) with each outcome versus not seeing a benefit (neither agree nor disagree, disagree or strongly disagree).

### Primary predictor variables: mistrust measures

The 21 items that constitute the medical mistrust measure were adapted from the Medical Mistrust Index, created and validated by LaViest and colleagues [32]. Three separate sub-scales to measure institutional medical mistrust in health care organizations, federal government, and local/state governments were created, specifically for the context of the COVID-19 pandemic. Each sub-scale contains seven statements with which participants were asked to choose their level of agreement, including statements such as “My community has sometimes been deceived or misled by [health care organizations / the local and state government / the federal government] regarding the COVID-19 pandemic” and “Mistakes are common in [health care organizations / the local and state government / the federal government] regarding the COVID-19 pandemic”. Each statement utilized a four-point Likert-scale, with responses ranging from one (strongly disagree) to four (strongly agree). The level of agreement for overall medical mistrust was measured by the average score (range 1–4) for all 21 statements, with higher values indicating a higher level of mistrust.

### Additional predictor variables

Binary predictor variables included gender (female vs. male), educational status (high school degree/some college vs. bachelor’s degree or higher), insurance status (insured vs. uninsured), employment status (employed vs. unemployed), marital status (not married vs. married), and regular access to a healthcare provider (yes vs. no). Worry of COVID-19 infection, described by the question “During the past two weeks, how worried have you been about being infected?”, was treated as a dichotomous variable with responses grouped into any level of worry (extremely, very, slightly, or moderately) vs. not worried. Age was measured as a continuous variable. Participants were asked to provide a self-reported history of nine comorbidities (yes = 1, no = 0). SCD severity was then measured by a composite score based on responses with higher scores indicating higher levels of SCD severity (range 0–8) [33]. SCD genotype was also collected to describe our population but was not included in final analysis models.

### Statistical analysis

Participant sociodemographic, behavioral, and clinical characteristics were assessed using descriptive statistics, including Chi-square test or Fisher’s exact test for categorical variables and two-sample t-tests for continuous variables. Frequencies, or mean and standard deviations (SD) for continuous variables, were identified for our outcome variables and independent covariates. Missing data were treated using listwise deletion and regression diagnostics were performed to examine outliers and influencers, including testing for model validity (i.e., homoskedasticity, variance inflation factor/multicollinearity, studentized residuals). Sensitivity analysis followed the identification of two outliers, which were removed upon ascertainment of influence. After removing missing values and outliers, 185 individuals were included in final analyses. Multivariable binary logistic regression models were utilized for the two outcomes, adjusting for applicable sociodemographic, behavioral, and clinical variables. Inclusion of covariates in final models was based on a significant association with each outcome during univariate diagnostic analysis. Race was not included as a covariate in final models due to majority of our sample population identifying as Black/African American. Statistical significance for all results were determined based on a p-value of < 0.05. All statistical analyses were completed using SAS version 9.4.

## Results

Descriptive univariate statistics are described in Table 1. A majority of study participants agreed with statements regarding beliefs about the importance of participating in vaccine research (71.4%) and the utility of vaccine research for family and self (60.0%), specifically in the context of COVID-19. The average level of medical mistrust was 2.9, with a score range of 1–4 (SD 0.60) (Table 1).

**Table 1:** Characteristics of sample population (*N* = 185)

	Total sample N (%)	Views on importance of people taking part in COVID-19 vaccine research			Views on the utility of COVID-19 vaccine research		
		Agree N (%)	No Opinion/Disagree N (%)	*p*-value	Agree N (%)	No Opinion/Disagree N (%)	*p*-value
**TOTAL**	185	132 (71.4)	53 (28.6)	-	111 (60.0)	74 (40.0)	-
**Age**				0.33			0.62
* Mean [Range]*	36.6 [18–68]	37.1 [18–68]	35.4 [19–67]		36.9 [18–68]	36.1 [19–67]	
* (SD)*	-10.7	-10.7	-10.7		-10.5	-11.1	
**Gender**				0.17			**0.01**
Female	115 (62.2)	78 (42.1)	37 (20.0)		61 (33.0)	54 (29.2)	
Male	70 (37.8)	54 (29.2)	16 (8.7)		50 (27.0)	20 (10.8)	
**Genotype**				0.88			0.29
HbSS	118 (63.8)	84 (45.4)	34 (18.4)		67 (36.2)	51 (27.6)	
HbSC	32 (17.3)	22 (11.9)	10 (5.4)		19 (10.3)	13 (7.0)	
Other Genotype ^a^	35 (18.9)	26 (14.0)	9 (4.9)		25 (13.5)	10 (5.4)	
Race*				**< 0.001**			**< 0.001**
American Indian/Alaska Native	1 (0.6)	-	1 (0.6)		-	1 (0.6)	
Black/African American	154 (85.6)	108 (60.0)	46 (25.6)		91 (50.6)	63 (35.0)	
Native Hawaiian/Pacific Islander	2 (1.1)	2 (1.1)	-		1 (0.6)	1 (0.6)	
White	16 (8.9)	15 (8.3)	1 (0.6)		16 (8.9)	-	
Other ^b^	7 (3.9)	7 (3.9)	-		2 (1.1)	5 (2.8)	
Ethnicity*				0.81			0.96
Not Hispanic/Latino	160 (88.9)	116 (64.4)	44 (24.4)		95 (52.8)	65 (36.1)	
Hispanic/Latino	20 (11.1)	14 (7.8)	6 (3.3)		12 (6.7)	8 (4.4)	
Income*				**0.01**			**0.01**
$59,999 or less	78 (43.8)	64 (36.0)	14 (7.8)		53 (29.8)	22 (12.4)	
$60,000 or greater	100 (56.2)	64 (36.0)	36 (20.2)		56 (31.5)	47 (26.4)	
**Education**							
High school Degree/Some College	81 (43.8)	56 (30.3)	25 (13.5)	0.56	43 (23.2)	38 (20.5)	
Bachelor’s or Higher	104 (56.2)	76 (41.1)	28 (15.1)		68 (36.8)	36 (19.5)	0.09
**Insurance Status**				0.44			0.58
Insured	170 (91.9)	120 (64.9)	50 (27.0)		101 (54.6)	69 (37.3)	
Not Insured	15 (8.1)	12 (6.5)	3 (1.6)		10 (5.4)	5 (2.7)	
Employment Status*				0.07			**0.01**
Employed	98 (55.7)	75 (42.6)	23 (13.1)		67 (38.1)	31 (17.6)	
Not employed	78 (44.3)	50 (28.4)	28 (15.9)		37 (21.0)	41 (23.3)	
**Marital Status**				0.32			0.19
Married	91 (49.2)	68 (36.8)	23 (12.4)		59 (31.9)	32 (17.3)	
Unmarried	94 (50.8)	64 (34.6)	30 (16.2)		52 (28.1)	42 (22.7)	
**Healthcare Provider** ^c^				0.1			0.46
Yes	173 (93.5)	126 (68.1)	47 (25.4)		105 (56.8)	68 (36.8)	
No	12 (6.5)	6 (3.2)	6 (3.2)		6 (3.2)	6 (3.2)	
**Worried about COVID-19 Infection**							
Any level of worry ^d^	155 (83.8)	117 (63.2)	38 (20.5)	**0.005**	99 (53.5)	56 (20.3)	**0.01**
Not worried	30 (16.2)	15 (8.1)	15 (8.1)		12 (6.5)	18 (9.7)	
**Sickle Cell Disease Severity Score**							
* Mean [Range]*,	2.9 [0–8]	3.1 [0–8]	2.4 [0–6]	**0.02**	3.3 [0–8]	2.4 [0–7]	**< 0.001**
* (SD)*	-2	-2	-1.8		-2	-1.8	
**Medical Mistrust**		2.9 [1.2-4] (0.5)		0.25			**0.03**
* Mean [Range]*	2.9 [1–4]		3.0 [1.9–3.9]		2.8 [1.2-4]	3.0 [1.9-4]	
* (SD)*	-0.6		-0.6		-0.6	-0.5	

^*a*^*Other genotype includes Hb Sß+-Thalassemia*,* Hb Sß0-Thalassemia*,* Sickle HPFH*,* Sickle Delta Beta (0) Thalassemia*,* Sickle HbO-Arab*,* Sickle HbE*

^*b*^ *Other includes: White/Hispanic or Other/Mixed*

^*c*^*Do you have a doctor or nurse you usually see if you need a check up*,* want advice about a health problem*,* or get sick or hurt?*

^*d*^*Any level of worry includes slightly*,* moderately*,* very*,* or extremely worried of infection*

* Totals reflect complete data after the removal of missing responses.

It is important for people to take part in COVID-19 vaccine research.

The unadjusted model between the importance of participating in COVID-19 research and medical mistrust was not statistically significant (OR = 0.71, 95% CI: 0.389–1.282, *p* = 0.25) (Table 2). Similarly, the association for this relationship continued to be non-significant after adjusting for relevant sociodemographic, behavioral, and clinical variables (OR = 0.52, 95% CI: 0.257–1.067, *p* = 0.07) (Table 2). Additionally, having any level of worry of COVID-19 infection, specifically within the last two weeks of study participation, was significantly associated with greater agreement with the importance of participating in COVID-19 vaccine research, after adjusting for relevant variables (OR = 3.41, 95% CI 1.346–8.641, *p* = 0.01) (Table 2).

**Table 2:** Unadjusted and adjusted multivariate regression models of independent variables and outcomes for vaccine research viewpoints (*N* = 185)

	Model 1: “Important for people to take part in COVID-19 vaccine research”		Model 2: “Vaccine research will help my family and me”	
	Unadjusted OR (95% CI)	Adjusted OR (95% CI)	Unadjusted OR (95% CI)	Adjusted OR (95% CI)
**Medical Mistrust**	0.71 (0.389–1.282)	0.52 (0.257–1.067)	**0.54** (0.310–0.951) *	**0.44** (0.222–0.890) *
**Gender** Female ^a^	–	–	–	1.62 (0.765–3.419)
**Income** $60,000 or greater ^d^	–	0.60 (0.257–1.408)	–	0.91 (0.403–2.045)
**Education** Bachelor’s Degree or Higher ^c^	–	–	–	0.77 (0.364–1.618)
**Employment Status** Employed ^e^	–	1.30 (0.580–2.920)	–	1.98 (0.89–4.395)
**Worried about COVID-19 Infection** Any level of worry ^b^	–	**3.41** (1.346–8.641) *	–	**3.54** (1.332–9.403) *
**Healthcare Provider** ^f^ Yes	–	2.59 (0.705–9.532)	–	–
**SCD Clinical Severity**	–	1.18 (0.968–1.433)	–	**1.26** (1.043–1.537) *

* p-value < 0.05

^*a*^ *Female vs. Male*

^*b*^*Any level of worry includes slightly*,* moderately*,* very*,* or extremely worried of infection vs. Not worried*

^*c*^ *Bachelor’s or higher vs. High School or Some College*

^*d*^*$60*,*0000 or greater vs. Less than $59*,*999*

^*e*^ *Employed vs. Not Employed*

^*f*^*Do you have a doctor or nurse you usually see if you need a checkup*,* want advice about a health problem*,* or get sick or hurt? Response of Yes vs. No*

Participation in COVID-19 vaccine research can help my family and me.

In the unadjusted model, each unit increase in medical mistrust is associated with a 46% decrease in the odds of agreeing with the utility of COVID-19 vaccine research (OR = 0.54, 95% CI: 0.31–0.951, *p* = 0.03) (Table 2). This association continued to be significant after adjusting for relevant sociodemographic, behavioral, and clinical variables (OR = 0.44, 95% CI: 0.222–0.89, *p* = 0.02) (Table 2). Additionally, higher self-reported worry of COVID-19 infection was significantly associated with higher agreement with the utility of vaccine research for themselves and their families, after adjusting for covariates (OR = 3.54, 95% CI: 1.332–9.403, *p* = 0.01). The adjusted model also indicated that agreement with this viewpoint was significantly associated with higher SCD severity (OR = 1.26, 95% CI: 1.043–1.537, *p* = 0.02) (Table 2).

## Discussion

Our study examined viewpoints of adults with SCD on COVID-19 vaccine research participation prior to the approval of vaccinations by the FDA and other regulatory agencies. We examined two different outcomes pertaining to (1) the importance of participation in COVID-19 vaccine research and (2) the utility of COVID-19 research participation for people and their families. The first investigated individuals’ viewpoints regarding the importance of people in general participating in vaccine research. Findings revealed that individuals with any level of self-reported worry of COVID-19 infection within two weeks of participating in the study expressed greater agreement with this statement. This relationship was maintained when examining individuals’ viewpoints on the utility of COVID-19 vaccine research participation for themselves and their families. Furthermore, agreement with the utility of COVID-19 vaccine research participation was also found to be associated with higher SCD severity, while higher medical mistrust was inversely associated with agreement of this statement.

The urgency of this pandemic and our finding that worry sentiments of becoming severely ill with COVID-19 among individuals living with SCD can be understood as the byproduct of individual-level vulnerability and perception of risk that people living with severe chronic illnesses may have. Individuals living with chronic illnesses report heightened levels of pandemic-related worry and self-perceived risk of severe infection compared to the general population [34, 35]. Existing literature has predominantly emphasized the role of worry of COVID-19 infection as a predictor of vaccination willingness and status, revealing that greater levels of worry are associated with more positive perceptions of the COVID-19 vaccine and increased vaccine uptake [36–38]. In 2020, a study striving to understand vaccine propensity in the U.S. found that shifting risk assessments on an individual level, such as disease severity, can be a driving factor for higher vaccine uptake within some communities [39]. Studies investigating worry of COVID-19 infection as it relates to perceptions of research participation and utility are minimal. Worry of infection has been cited as a barrier to personal research participation in vulnerable subgroups of Black/African American communities due to increased fear of exposure to the virus in clinical research settings, but overall perceptions of the importance and utility of the research remained positive among these study populations [40, 41]. Our finding that medical mistrust was inversely associated with viewpoints on the utility of COVID-19 vaccine research participation further emphasizes the gap in understanding the role that medical mistrust has on individuals living with a chronic disease and their perceptions and decisions to participate in clinical trial research on a grander scale.

While the historical context of unethical research performed on communities of color is often cited as a cause of high mistrust among individuals who identify as Black/African American, our study findings present a different narrative. Particularly, we found that the views of individuals living with sickle cell disease are fundamentally tied to their concerns of risks posed by the COVID-19 pandemic, such as worry of infection and disease severity, more so than it being solely related to mistrust. Daly and colleagues found that mistrust is correlated with vaccine hesitancy in communities of color [42]. However, the authors also found that vaccine uptake among Black and Hispanic communities increased over time at higher rates than for White participants, further offering new perspective to the discourse on hesitancy and mistrust among racial and ethnic minority communities [42]. Related to that study, our findings also illustrate how the typical generalized discourse of sentiments of mistrust within racial and ethnic minority communities fails to capture the other considerations individuals may have in participating in COVID-19 vaccine research, especially when faced with higher health risks. Therefore, consistent with a growing perspective in the era of COVID-19, nuance is required in the way we frame mistrust [43]. Furthermore, scholars are drawing more attention to how the narrative of mistrust can place blame on individuals from communities of color and excuse structurally-produced barriers that fail to provide access to vaccines and antiviral therapies in underserved communities [44–46]. For example, scholars have found that while historical mistrust may cause some hesitation about research participation, if given the opportunity to learn more about a clinical trial or research study, many individuals of racial and ethnic minority communities would be open to participating, especially if it would benefit their health, well-being, or community [8, 47].

Thus, ongoing efforts to vaccinate hesitant individuals call for a better understanding of the social, psychological, and clinical factors associated with vaccine research participation and uptake, especially among individuals living with chronic diseases like SCD. Furthermore, given that the SCD population has unique experiences at the intersection of race and illness, our findings build on research that has found that the aspects of the SCD lived experience, like pain and social identity, impact research participation [48]. We identify additional considerations that individuals with an underlying racialized medical condition deem important, specifically how worry of COVID-19 infection and disease severity impact views towards vaccine research.

The study limitations include potential recruitment bias as individuals without internet access were unable to participate, and therefore, were not surveyed. Rather, participants were recruited through a current NIH-funded SCD study, advocacy groups, and social media. As the study population contained a majority of highly educated individuals living above the national poverty level who had low disease severity scores, the viewpoints of individuals with low socioeconomic status and higher disease severity are missing. Since this was a self-administered survey, veracity of participants responses was assessed by response consistency. Additionally, this study was limited by a small, non-random sample size of 185 participants living with SCD. While this challenges the generalizability of our findings, it also allows for a sharper exploration of the experience of living with a rare and chronic disease during a pandemic. Finally, this study is limited by the fact that the science, policy, and medicine surrounding COVID-19 are fast-moving and have inconsistently changed throughout the pandemic, specifically during the time of this study.

## Conclusion

It is critical to assess the attitudes and sentiments of individuals living with chronic conditions on participation in vaccine research. Individuals living with chronic diseases face many challenges in managing their health outside of a global pandemic. Chronic diseases like SCD pose challenges and risks throughout other crises, such as housing insecurity, food insecurity, and structural racism in health care [49]. In the post COVID-19 pandemic era, we seek to draw lessons on how to better prioritize and comprehensively measure the attitudes and views of individuals living with chronic genetic conditions in vaccination research and uptake.

## Data Availability

The datasets generated and/or analyzed during the current study are available from the corresponding author on reasonable request.

## Abbreviations

FDA: Food and Drug Administration
NIH: National Institutes of Health
OR: Odds ratio
SCD: Sickle cell disease
SD: Standard deviation
